# COVID-19 Policy Differences across US States: Shutdowns, Reopening, and Mask Mandates

**DOI:** 10.3390/ijerph17249520

**Published:** 2020-12-18

**Authors:** Xue Zhang, Mildred E. Warner

**Affiliations:** 1 Department of City and Regional Planning, Cornell University, Ithaca, NY 14850, USA; mwarner@cornell.edu; 2 Department of Global Development, Cornell University, Ithaca, NY 14850, USA

**Keywords:** COVID-19, state policy, shut down, reopen, mask mandate, Medicaid expansion, essential worker, minority

## Abstract

This work used event study to examine the impact of three policies (shutdowns, reopening, and mask mandates) on changes in the daily COVID-19 infection growth rate at the state level in the US (February through August 2020). The results show the importance of early intervention: shutdowns and mask mandates reduced the COVID-19 infection growth rate immediately after being imposed statewide. Over the longer term, mask mandates had a larger effect on flattening the curve than shutdowns. The increase in the daily infection growth rate pushed state governments to shut down, but reopening led to significant increases in new cases 21 days afterward. The results suggest a dynamic social distancing approach: a shutdown for a short period followed by reopening, combined with universal mask wearing. We also found that the COVID-19 growth rate increased in states with higher percentages of essential workers (during reopening) and higher percentages of minorities (during the mask mandate period). Health insurance access for low-income workers (via Medicaid expansion) helped to reduce COVID-19 cases in the reopening model. The implications for public health show the importance of access to health insurance and mask mandates to protect low-income essential workers, but minority groups still face a higher risk of infection during the pandemic.

## 1. Introduction

On 11 March, the WHO declared COVID-19 as a pandemic. By the end of August 2020, 6,045,455 people were infected in the US, and 183,472 had died [1]. The pandemic has disproportionally affected minority groups, low-income families, and essential workers in the US [2].

The rapid spread of COVID-19 required a quick government response. Social distancing practices are the most efficient way to reduce COVID-19 spread and were implemented in China [3], South Korea [4], Singapore [5], the UK [6], Spain [7], and many countries in the European Union [8]. In the US, state governments took the lead in responding to the pandemic. On 19 March 2020, California was the first state to impose a stay-at-home order to slow the spread. The efficiency of social distancing practices shows up in the infection curve. In the first wave of the pandemic in the US, the daily new cases peaked in mid-April (32,000/day) and then slowly decreased [1]. However, the soaring unemployment rate in April and May pushed states to reopen too soon. South Carolina was one of the first states to reopen, only 13 days after its stay-at-home order was first imposed. With the economy starting to open up, daily new cases dramatically increased, especially in the southern and western states, producing a much higher second wave in mid-July (80,000/day) [1]. The new case rate dropped after August, as more states issued mandated facial masking orders. New York State was the COVID-19 epicenter back in April 2020, but due to its early stay-at-home order, mandated facial mask wearing, and later reopening, its new case rate dropped to around 2% (14-day average) in August 2020, compared to 12% nationwide [1]. By contrast, Florida and Texas, with early reopening, had more cumulative cases than New York in August 2020. By the end of August 2020, the top five states (CA, TX, FL, NY, and GA) accounted for 44% of total COVID-19 cases. This study used event analysis to dynamically analyze the relation between state policy and the daily COVID-19 infection growth rate during the first two waves of the pandemic in the US (February through August 2020).

In the US, state governments play an important role in providing services and social citizenship rights [9,10]. This has been especially true during COVID-19, where the federal government left COVID-19 policy responses primarily to the states [10,11,12]. Under the Affordable Care Act enacted in 2010, states had the opportunity to expand Medicaid to address the high uninsured rates among low-income residents. This federal government offered states opportunities to design and implement the expansion of access to health insurance through Medicaid [13]. As of November 2020, 36 states offered expanded Medicaid access [14]. For minority populations, the enactment of the Affordable Care Act and expanded Medicaid have led to a higher share of insurance coverage [15,16].

This study examined the relation between the daily growth rate in COVID-19 infection (7-day rolling average) and three state-level polices in the US: shutdowns, reopening, and mask mandates. We analyzed the change in the COVID-19 infection growth rate before and after policy implementation and used dynamic event modeling to capture this relation over time. We were also interested in the role of Medicaid expansion on the COVID-19 spread, and the relation between the demographic structure (essential workers and minority population) and COVID-19 infection growth rate in each policy period. This study addressed the following questions: Which policies are more responsive to an increase in COVID-19 infection growth rates, and which have the greatest impact on reducing the COVID-19 infection growth rate? How is the COVID-19 infection growth rate related to Medicaid expansion, essential workers, and minority groups?

### Literature Review

Social distancing measures play an important role in COVID-19 prevention. A global-level study found that policies reducing contact among individuals, such as business/school closures and no gatherings, prevented 61 million cases across China, South Korea, Italy, Iran, France, and the US by the end of March 2020 [17]. A study of 340 cities in China predicted that a three-week-earlier intervention of travel restrictions and isolation could have reduced COVID-19 cases by 99%, while the cases would have been 67 times higher without the intervention [18]. Similar results have been estimated in the US: one study found that imposing stay-at-home orders one week earlier could have prevented 61% of infections and 55% of deaths by 3 May 2020 [19].

Although the shutdowns helped to reduce the COVID-19 infection rate, they also led to a global economic recession [20]. Poverty and social inequity significantly increased during the social distancing and lockdowns in European countries [21]. In the US, most states imposed stay-at-home orders in March and April 2020, and the national unemployment rate peaked in April at 14.44% and stayed above 10% through August 2020 [22]. Unemployment is the highest among low-income workers and minority families [23]. Although social distancing resulted in losses to GDP, research shows that the cost to GDP could be offset by the value of the lives saved [24]. Some states in the US reopened when cases were still rising [25]. By 1 June 2020, every state had reopened at least one sector [26].

With the lifting of shutdown orders, mask wearing became the primary way to slow the COVID-19 spread around the world [8,27,28,29,30]. Early studies in New York and Washington showed the efficiency of universal mask wearing in reducing the community transmission of COVID-19, even for homemade and relatively low-quality masks [28]. However, up to September 2020, 14 out of 50 states in the US still did not mandate mask wearing in public [31]. The lifting of shutdown orders and the rapid move to reopening disproportionally affected the minority population, as a higher percentage of minorities work in low-paid essential occupations [2,32], and minority communities have less access to health care and prevention, and a low quality of health care generally [33,34]. Due to inequities in the social determinants of health among minority groups, the infection rate among minorities is almost three times the infection rate of whites [35].

The US states’ responses to COVID-19 provide a unique case study on the role subnational governments play in the pandemic. In contrast to other countries, such as Mainland China, Taiwan, and South Korea, where centralized strategies were implemented to control the COVID-19 spread, in the US, state governments were expected to take the lead [10,11,36]. The cases in the EU show that sound and strong leadership, and proper and quick decisions are essential for responding to the pandemic, and this requires collaboration from governments at all levels [8]. However, in the US, the federal government mis-stepped in the early stage of the pandemic in testing and delivering information, and in providing guidelines and suggestions for states to follow [25,36]. The US became the new epicenter of COVID-19, in part due to the lack of strong federal leadership [36] and the variation in states’ actions. The variation in the implementation dates of state policies and in the resulting COVID-19 infection growth rates provides an opportunity to assess the effectiveness of the three different policies over time and provide insights for future government action as we enter the third wave of the pandemic in the US.

## 2. Methods

### 2.1. Data

#### 2.1.1. Outcome Variable: Daily Infection Growth Rate

This study explored the relation between the state-level daily COVID-19 infection growth rate and the date when each state imposed each of the three policies. The daily growth rate is used in other COVID-19 studies to measure the relation between policy implementation and COVID-19 infections [27,37,38]. The daily growth rate was measured using the 7-day rolling average of daily new confirmed cases. The calculation is shown below.

The 7-day average infections on *day i* are:

(1) $${Infection}_{day\;i}=\;\frac{\sum_{i-3}^{i+3}the\;number\;of\;new\;confirmed\;cases\;\left(new\;tests\right)\;on\;day\;i}{7}$$

*Infection_day i_* is used to calculate the growth in the 7-day average COVID-19 infections on *day i*:

(2) $${Infection\;Growth}_{day\;i}\;=\;\frac{Infection\;day\;i}{\;Infection\;day\;i-1}\;-\;1$$

We used the same approach to calculate the 7-day average growth in tests.

#### 2.1.2. Independent Variables

##### Policy Implementation

Three policies were examined in this study: shutdowns, reopening, and mask mandates. The New York Times [26] tracks the dates of states’ shutdown and reopening policies. California was the first state to impose a stay-at-home order on 19 March 2020, followed by Illinois, New Jersey, and New York. South Carolina was the last state to impose a stay-home order during the first wave, on 7 April 2020. Eight states never imposed a shutdown order. States gradually reopened, beginning in late April 2020. This study used the earliest sector reopening date from the New York Times database [26] to represent the states’ reopening dates. South Carolina was the first state to reopen retail stores on 20 April 2020, followed by Texas and Georgia. Nebraska was the last state to reopen the sectors of food and drink and personal care on 1 June 2020. Although eight states did not impose a statewide shutdown order in the first wave, seven published a reopening date, and in the eighth, South Dakota, the governor said the state would “get back to normal” on 28 April 2020 [39].

CNN tracks the state orders requiring mandated mask wearing [31]. By the end of August 2020, 36 out of 50 states required people to wear a mask in the public. New Jersey was the first state to impose a mandated masking order on 8 April 2020, 18 days after the shutdown order on 21 March and 39 days before the reopening order on 18 May 2020. New Hampshire was the last state to impose a mask mandate in the time period of our analysis, on 11 August 2020, three months after its reopening order.

**Figure 1:**
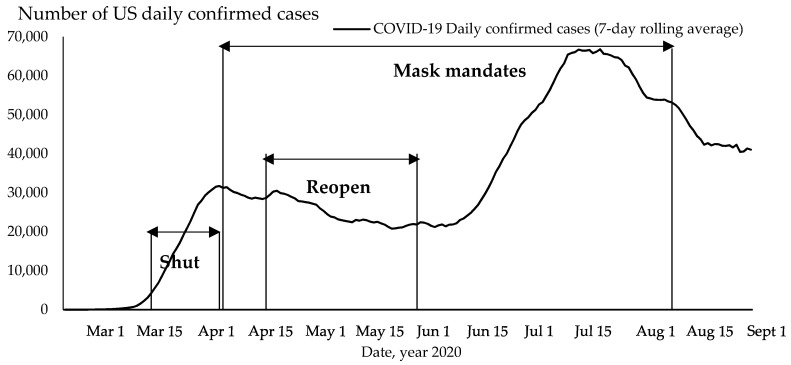
Policy implementation period and COVID-19 daily new infections, US. Note: earliest policy start date to latest policy start date across 50 US states. Source: author’s analysis of state policy using NY Times [1,26] and CNN data [31].

Figure 1 shows the national daily COVID-19 new confirmed cases (7-day rolling average) and the policy duration between the earliest policy start date and the latest start date across the 50 states (see Appendix A for detailed information on each state). Shutdown orders were imposed during the first wave, and reopening orders were issued when the national confirmed case rate slowly decreased after the first peak in April 2020. The COVID-19 daily infection growth dramatically increased after reopening and led to a second wave, which peaked in July 2020. The date of issuing mask mandates also varied widely across the states, beginning with New Jersey, which was the first to implement a mask mandate during the first wave in April 2020.

The fluctuation of COVID-19 daily infections (shown in Figure 1) illustrates the dynamic impact of policy intervention on the national spread of the epidemic. Shutdown orders were an early response to the exponential growth of COVID-19 infections in early March 2020, but the orders were replaced by reopening policies starting at the end of April. Reopening was implemented as a response to the surge in unemployment in April 2020, but the COVID-19 daily infection once again showed exponential growth at the end of June 2020. The mask mandate orders were imposed in response to the high infection increases during the first and second waves. The different dates and durations of the state policies created opportunities for comparative study.

This study selected a three-week period before the earliest policy implementation date and a three-week period after the latest policy implementation date for each policy (Table 1). The three-week period can capture the relation between policy implementation and daily infection fluctuation. For the shutdown order, the three-week period covered the beginning of the pandemic and the beginning of the reopening orders. For the reopening orders, the study period covered the high infection growth in the first wave and the decrease in daily infections before the second wave. For the mask mandates, the three-week period covered the first peak and the exponential growth of COVID-19 infections during the second peak. The three-week period provided enough variation in infection rates for the policy data, and allowed a comparison across states in terms of their policy implementation.

Table 1 shows that the 7-day average daily COVID-19 infection growth rate was the highest in the shutdown period (14.35%), as most states imposed the order during the early months of the first wave. The reopening period had a lower average daily infection growth rate (Table 1, 1.38%), as the COVID-19 spread gradually slowed down after the shutdown. The mask mandate period spanned the first and the second waves, and the entire period had an average 2.74% daily COVID-19 infection growth rate. The average daily test growth rate had a similar trend, dramatically increasing (30.16% on average) at the beginning of the pandemic (during the shutdown period as states increased their testing capacities) and with lower rates of increase (4.39%) during reopening, because expanded testing provided a higher base and a higher rate in the second wave—an average 5.7% daily growth in tests.

#### Other Independent Variables

In addition to the three state policies of primary interest, we were also interested in the effects of Medicaid expansion (to cover more low-income residents) on the rate of COVID-19 spread. Thirty-four of 50 states had adopted Medicaid expansion during the period of our study [14]. If a state had adopted Medicaid expansion, we coded it as 1.

Essential workers and minority groups are some of the most vulnerable populations in the pandemic [2,32]. For each policy, we were interested in the relation between the COVID-19 daily infection growth rate and the percentage of essential workers and minorities. The Center on Budget and Policy Priorities (CBPP) calculated the number of essential workers for each state, including those working in “essential food production, essential manufacturing (including medicine), essential public services (including civic and public safety), essential transportation, essential utilities, essential warehousing, front-line health care services, front-line retail, and front-line services (including transportation and child care)” [40]. We divided the number of essential workers by the employed population aged 16 years and over for each state. Minorities were calculated as 1 minus the percentage of non-Hispanic whites, drawn from the American Community Survey (2014–2018). We also controlled for the daily test growth rate, which was calculated using the 7-day rolling average of daily tests. Descriptive statistics are shown in Table 1.

### 2.2. Methodology

This study used event study to assess the relation between the daily growth rate of COVID-19 infections and the date of policy implementation. We controlled for Medicaid expansion, vulnerable populations (essential workers and the minority population), and the daily test growth rate in each model. The regression model is shown below:

Daily (7-day average) infection growth rate (*Infection Growth_day i_*) = f {policy implementation (8 dummies for time before and after policy enactment), daily test growth rate (7 day average), percentage of essential workers, percentage of minorities, Medicaid expansion}.

We built panel data including all 50 states. For each state, we included time series data on the 7-day rolling average growth rates in COVID-19 infections and tests for each day during the study period. The time series data also included 8 independent variables in each of the three models to measure 8 points in time to capture the periods before and after when each of the three policies was implemented across the US states. There were 4 dummy variables measuring the pre-policy time period: whether the enactment date was more than 16 days, 11–15 days, 6–10 days, or 1–5 days before the policy was implemented. There were also 4 dummy variables measuring the post-policy time period: whether the date was 6–10 days, 11–15 days, 16–20 days, or more than 21 days after the policy was implemented. The reference group was the time period of policy implementation (enactment date, 0–5 days). For states that never imposed the policy, all the policy variables were set to 0. Table 2 uses New York State as an example to show the data structure in the shutdown model (the study period is from 27 February 2020 to 28 February 2020, Table 1). New York imposed the shutdown order on March 22, so the dates before 22 March 2020 were captured in the pre-policy variables, and the dates after 22 March 2020 were captured in the post-policy variables (Table 2). South Dakota did not impose a shutdown order, so the policy implementation variables were all zero.

Event study fitted this research because the event model could detect both the time lag of policy implementation and the reverse-causal relation between COVID-19 cases and future policy [37]. Event study could estimate the relation between the COVID-19 daily infection growth rate in the pre-policy period to explore if the growth in the COVID-19 infection rate encouraged state governments’ actions. In addition, event study could examine the relation to the COVID-19 infection growth rate in the post-policy period to explore the impact of policy implementation on COVID-19 spread.

## 3. Results

We ran three event study models in STATA 14 to examine the factors related to the COVID-19 daily infection growth rate over the study period (Table 1) for three policies: the implementation of shutdowns, reopening, and mask mandates. The models were estimated using multilevel mixed-effects linear regression nested at the state level. We used the multilevel model because that reflects the structure of our data. The model results are shown in Table 3. The coefficients were standardized to compare the magnitude of the independent variables.

Table 3 shows the impact of policies on the COVID-19 infection growth rate. The shutdown order was significantly related to the daily infection growth rate during the time period, both pre-policy and post-policy. The coefficients were standardized and show the standard deviation of the impact of the policy on the COVID-19 infection growth rate. Before the shutdown order was imposed, the COVID-19 daily growth rate was increasing by 0.15 std. dev. (16 days before), 0.19 std. dev. (11–15 days before), 0.13 std. dev. (6–10 days before), and 0.06 std. dev. (1–5 days before). After the shutdown order was imposed, the daily growth rate declined by 0.04 std. dev. (6–10 days after), 0.07 std. dev. (11–15 days after), 0.08 std. dev. (16–21 days after), and 0.1 std. dev. (21+ days after). The model results show that the COVID-19 infection growth rate peaked within 11–15 days before the order and then declined after the order.

**Figure 2:**
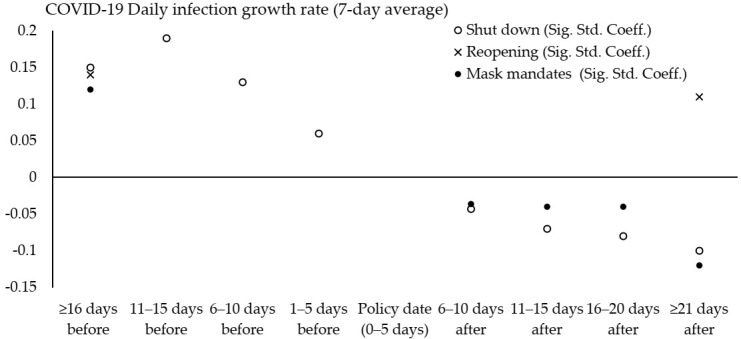
Relation between state policies and COVID-19 daily infection growth rate before/after implementation. Source: significant coefficient results from Table 3; 50 US states; February through August 2020.

Figure 2 shows the significant coefficients from Table 3 to illustrate the relation between policy implementation and the COVID-19 infection growth rate over time. The reopening order was not significantly related to the COVID-19 daily infection growth rate, except 16 days before the order and 21 days after (Figure 2). The COVID-19 infection growth rate increased by 0.14 standard deviations 16 days before the reopening order and increased by 0.11 standard deviations 21 days after the reopening. Table 3 shows that states with a higher growth in daily tests also had a higher growth in infections during the reopening order (std. coeff. = 0.04) and mask mandate periods (std. coeff. = 0.08).

After states imposed the mask mandate order, the COVID-19 daily infection growth rate declined by 0.04, 0.04, 0.04, and 0.12 standard deviations, respectively, for the periods 6–10 days, 11–15 days, 16–20 days, and 21 or more days after the order (Table 3 and Figure 2). There was not a significant relation to the daily infection growth rate in the pre-mask mandate time period, except an increase in the infection growth rate by 0.12 standard deviations 16 days before the order. Figure 2 shows that after states imposed the order, shutdown orders had a larger impact on the decline in the COVID-19 infection growth rate than the mask mandates within the three week period. However, mask mandate orders had a larger negative effect after three weeks.

The impact of Medicaid expansion and the demographic structure on the COVID-19 daily infection growth rate varied across the three policies. None of these variables were significantly related to the COVID-19 infection growth rate in the shutdown model (Table 3). However, in the reopening model period, states that adopted Medicaid expansion had a lower COVID-19 infection growth rate, by 0.04 standard deviations, but a higher COVID-19 infection growth rate if they had more essential workers (std. coeff. = 0.03) (Table 3). A one standard deviation increase in the percentage of essential workers is related to a 0.04 standard deviation increase in the COVID-19 infection growth rate, but the impact of Medicaid expansion (Coeff. = −0.04) could offset this effect. In the mask mandate model period, states with a higher percentage of minorities had a 0.04 standard deviation increase in the COVID-19 daily infection growth rate, while Medicaid expansion and the percentage of essential workers were not significant (Table 3).

## 4. Discussion

Social distancing practices are the main public health response to COVID-19. States imposed shutdown orders at the beginning of the pandemic and then moved to reopen and to impose mask mandates (Figure 1 and Appendix A). This study confirms previous studies on the efficiency of shutdowns [37,38] and mask wearing [8,27,28,29,30] for controlling COVID-19 spread after states imposed the orders. We found that a rise in daily confirmed cases pushed states to impose shutdown orders but had no effect on states’ decisions to impose mask mandates. This shows that the growth in daily confirmed cases pressured state governments to act and impose shutdown orders. Shutdown orders play an important role in reducing the infection growth rate, but our results show that mask mandates have a larger effect in reducing COVID-19 infection growth rates than shutdowns after 21 days.

This study found that reopening orders resulted in an increase in COVID-19 infection growth rates 21 days after the orders were imposed. Other studies on policy implementation show that US states’ reopening orders were not related to dropping case rates, as would be expected for a public health measure, but rather were related to political partisanship at the state level (Republican control) [10]. Reopening is more likely to be a political response to the high unemployment rate, due to the shutdown, rather than a public health response to a drop in COVID-19 infections. Indeed, some states reopened when COVID-19 cases were rising, while other states waited until their cases were falling [11,12]. This is why the pre-policy implementation dates were not significant in our reopening model; the two types of state responses cancelled each other out.

Globally, research finds a dynamic approach to social distancing practices: a limited time of shutdown followed by reopening. A global-level study of 16 countries showed that a 50-day suppression followed by 30-day relaxation could significantly lower COVID-19 mortality rates [42]. Similar results were found in Canada [43], the UK, and China [44]. However, in the US, the politicization of the public health response made reopening less effective [10].

Reopening orders and mask mandates overlap in time. In an alternative model specification (not shown), we combined the reopening model and mask mandates to examine the relation between daily infection growth and policy implementation during the second wave. The results were similar to our original models: the daily infection growth rate increased during reopening and decreased after states imposed mask mandates. We also found that the infection growth rate increased in states with more essential workers and minorities (see Appendix B for the model results).

Our study confirms that essential workers and minority populations are more vulnerable during the pandemic [2,32,45]. What we found interesting is that Medicaid expansion could help during the reopening period, when essential workers face a higher risk of COVID-19 exposure. Data from the Center on Budget and Policy Priorities also show that states with Medicaid expansion have a higher percentage of essential workers enrolled in Medicaid (12% vs. 5% in non-expansion states) and cover a higher percentage of low-income essential workers (37% vs. 15% in non-expansion states) [40]. To further explore the role of Medicaid in COVID-19 spread during the reopening period, we ran two separate reopening models (not shown). These model results confirm that the COVID-19 daily growth rate declined in states with a higher percentage of essential workers enrolled in Medicaid (std. coeff. = −0.04, *p* < 0.05) and a higher percentage of low-income essential workers enrolled in Medicaid (std. coeff. = −0.06, *p* < 0.01).

The mask mandate model covers a longer study period, from 18 March to 1 September 2020 (Table 1). The results show that states with a higher percentage of minorities had a higher increase in daily COVID-19 infection growth rates. While the mask mandates protect essential workers, the higher case rates in states with more minority population can be explained by the social determinants of health. While the reopening model shows the importance of Medicaid expansion, states without Medicaid expansion have a significantly higher percentage of minority population than states with Medicaid expansion (33% vs. 30%, *p* < 0.05). A California study found that having insurance coverage from Medicaid does not guarantee access to primary care, which is related to broader structural inequalities [16]. The CDC has found that inequities in the social determinants of health help to explain the higher COVID-19 infection rates among minority groups [35]. The CDC encourages health care providers, public health agencies, and policy makers in minority communities to collaborate to increase access to information, testing, and medical and mental health care [35]. Collaboration between agencies has been shown to increase service availability in US communities, especially for older adults [46,47,48]. The United Nations High Commission on Human Rights points to some promising practices to address the disproportional impact of COVID-19 on minority populations worldwide, such as allocating aid in Greece, urgent measures for food solidarity in Italy, access to public services in Portugal, actions on social services in Spain, providing protection measures in the UK, etc. [32].

This study has several limitations. First, the reopening date was measured as the earliest date of sector reopening. It may not fully capture the reopening scenario. Future studies could explore the relation between the reopening date in different sectors and the spread of COVID-19. Second, this study focused on shutdown orders during the first wave of the pandemic in the US. Some states imposed shutdown orders again, due to the increase in new cases in November and December 2020—the third wave. Future studies could examine the relation between shutdown orders and COVID-19 spread in the third wave. Third, this study used the executive order as the measure of shutdown policy. Policies can lead to changes in behavior, such as a change in people’s mobility, which can be used to measure the level of shutdown [20]. Future research could examine the effect of shutdown orders on people’s behavior and link that to COVID-19 infection rates. Additionally, this study examined the relation between COVID-19 infections and policy implementation in the first and second waves. Future studies could examine the relation to the death rate over time. For example, Lyu and Wehby’s [38] research shows that shelter-in-place policies reduced mortality and hospitalizations in the US.

## 5. Conclusions

This study shows the importance of early invention in controlling the COVID-19 spread by assessing the impact of state-level shutdown orders and mask mandates on COVID-19 infection growth rates. Rising infection growth rates pushed state governments to impose shutdown orders, and these orders were effective in reducing infections. Reopening was unrelated to infection rates, but resulted in increased daily infection rates after 21 days. Mask mandates led to reduced infection growth rates after implementation. Our dynamic event study suggests a combination of interventions is the most effective policy response. Shutdown followed by reopening, combined with universal mask wearing, helps to lower the COVID-19 infection growth rate. This dynamic policy approach could potentially balance economic disruption and public health. This research also shows the importance of health insurance coverage, achieved through Medicaid expansion, especially for essential workers. However, structural inequality and health disparities in the social determinants of health contribute to a higher risk for minority groups. By conducting event analysis of three common pandemic response policies across the fifty US states, this study contributes to our global understanding of the importance of subnational policy differences. It also points to the need to expand health insurance coverage and address the structural inequalities that lead to higher infection rates in communities with more minority and essential workers.

## Figures and Tables

**Table 1:** COVID-19 state policy and descriptive statistics: 50 US states.

	Shutdown	Reopening	Mask Mandates
Outcome variable			
Daily COVID-19 infection growth rate (7-day rolling average, %) ^1^	14.35	1.38	2.74
Independent variables			
Policy implementation			
Number of states imposing the order ^2,3^	42	50	36
Earliest policy implementation date ^2,3^	19 March 2020	20 April 2020	8 April 2020
Latest policy implementation date ^2,3^	7 April 2020	1 June 2020	11 August 2020
Study period (21 days before to 21 days after policy implementation)	27 February 2020– 28 April 2020	30 March 2020– 22 June 2020	18 March 2020– 1 September 2020
Number of days	62	86	169
Other independent variables			
Daily test growth rate (7-day rolling average, %) ^1^	30.16	4.39	5.70
Medicaid expansion (1 = yes) ^4^	0.72	0.72	0.72
Percentage essential workers ^5,6^	32.90	32.90	32.90
Percentage minorities ^6^	30.89	30.89	30.89
Number of observations (number of days ×50 states)	3100	4300	8450

Source: ^1,2^ NY Times [1,26], 2020. ^3^ CNN [31], 2020. ^4^ KFF [14], 2020. ^5^ CBPP [40], 2020. ^6^ ACS [41] (2014–2018).

**Table 2:** Data structure: an example of New York State in the COVID-19 shutdown model.

Policy Implementation Variables										
Dates Before	Pre-Policy Variables				Policy Enactment Date, 0–5 Days	Dates After	Post-Policy Variables			
	≥16 Days	11–15 Days	6–10 Days	1–5 Days			6–10 Days	11–15 Days	16–20 Days	≥21 Days
					22 March 2020–27 March 2020					
27 February 2020	1	0	0	0		28 March 2020	1	0	0	0
…						…				
6 March 2020						1 April 2020				
7 March 2020	0	1	0	0		2 April 2020	0	1	0	0
…						…				
11 March 2020						6 April 2020				
12 March 2020	0	0	1	0		7 April 2020	0	0	1	0
…						…				
16 March 2020						11 April 2020				
17 March 2020	0	0	0	1		12 April 2020	0	0	0	1
…						…				
21 March 2020						28 April 2020				

Data: author’s analysis.

**Table 3:** COVID-19 US state policies: event study model results, February through August 2020 (standardized coefficients).

	Shutdown ^2^		Reopening ^2^		Mask Mandates ^3^	
	Daily Infection Growth Rate ^1^ (%)		Daily Infection Growth Rate ^1^ (%)		Daily Infection Growth Rate ^1^ (%)	
	Std. Coeff.	*p* Value	Std. Coeff.	*p* Value	Std. Coeff.	*p* Value
Daily test growth rate ^1^ (%)	0.03	(0.06)	0.04 **	(0.00)	0.08 **	(0.00)
Policy implementation						
Pre-policy period						
≥16 days before	0.15 **	(0.00)	0.14 **	(0.00)	0.12 **	(0.00)
11–15 days before	0.19 **	(0.00)	0.01	(0.69)	−0.00	(0.91)
6–10 days before	0.13 **	(0.00)	−0.00	(0.94)	−0.02	(0.10)
1–5 days before	0.06 **	(0.00)	−0.01	(0.75)	−0.02	(0.13)
Policy date (reference, 0–5 days)						
Post-policy period						
6–10 days after	−0.04 *	(0.02)	−0.00	(0.95)	−0.04 **	(0.00)
11–15 days after	−0.07 **	(0.00)	0.00	(0.81)	−0.04 **	(0.00)
16–20 days after	−0.08 **	(0.00)	0.02	(0.30)	−0.04 **	(0.00)
≥21 days after	−0.10 **	(0.00)	0.11 **	(0.00)	−0.12 **	(0.00)
Medicaid expansion ^4^ (1 = yes)	0.02	(0.29)	−0.04 *	(0.01)	0.02	(0.33)
Percentage essential workers ^5,6^	−0.01	(0.63)	0.03 *	(0.04)	0.02	(0.18)
Percentage minorities ^6^	0.00	(0.87)	0.02	(0.34)	0.04 *	(0.01)
N	3100		4300		8450	
Log likelihood	−1249.20		3567.05		6444.67	
Pseudo R	0.11		0.02		0.04	

Source: ^1,2^ NY Times [1,26], 2020. ^3^ CNN [31], 2020. ^4^ KFF [14], 2020. ^5^ CBPP [40], 2020. ^6^ ACS [41] (2014–2018). Note: * *p* < 0.05, ** *p* < 0.01.

**Figure A1:**
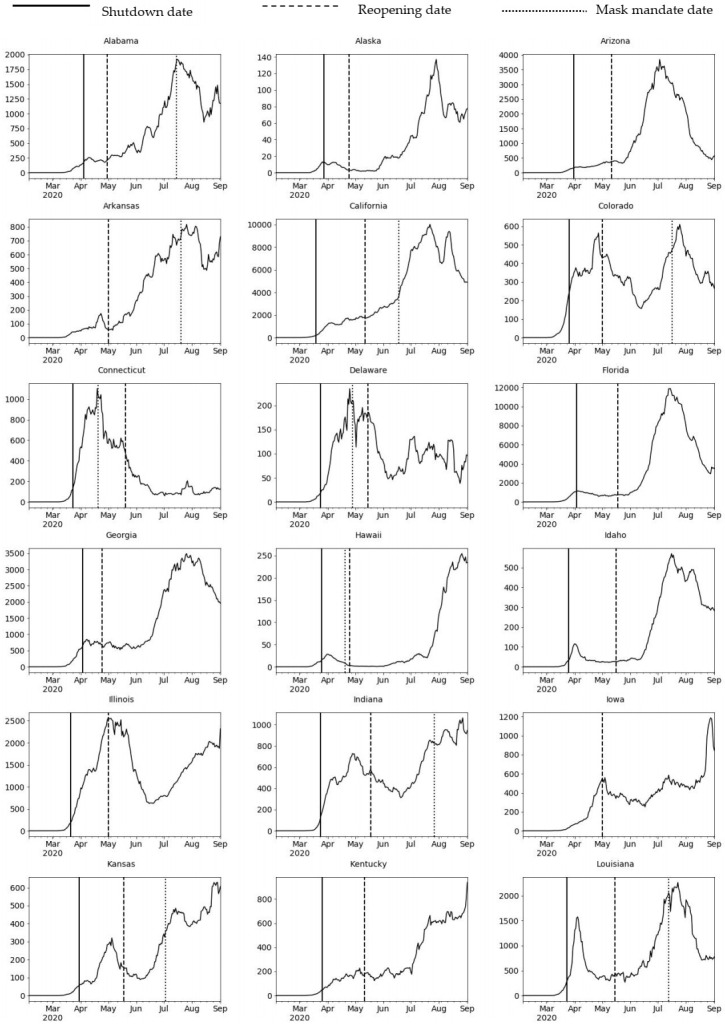

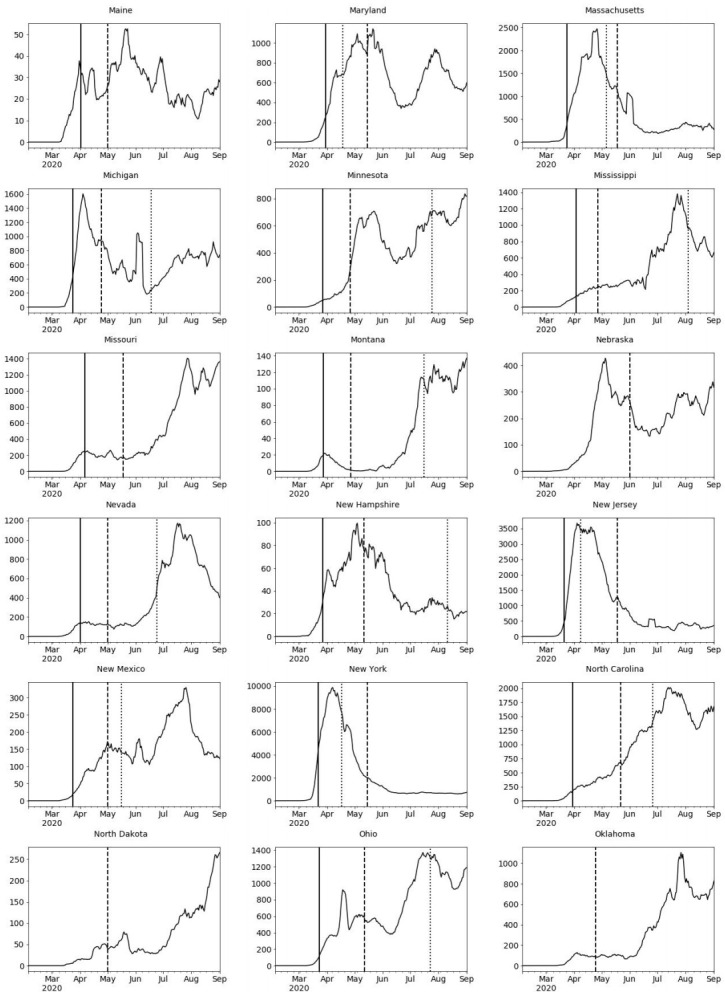

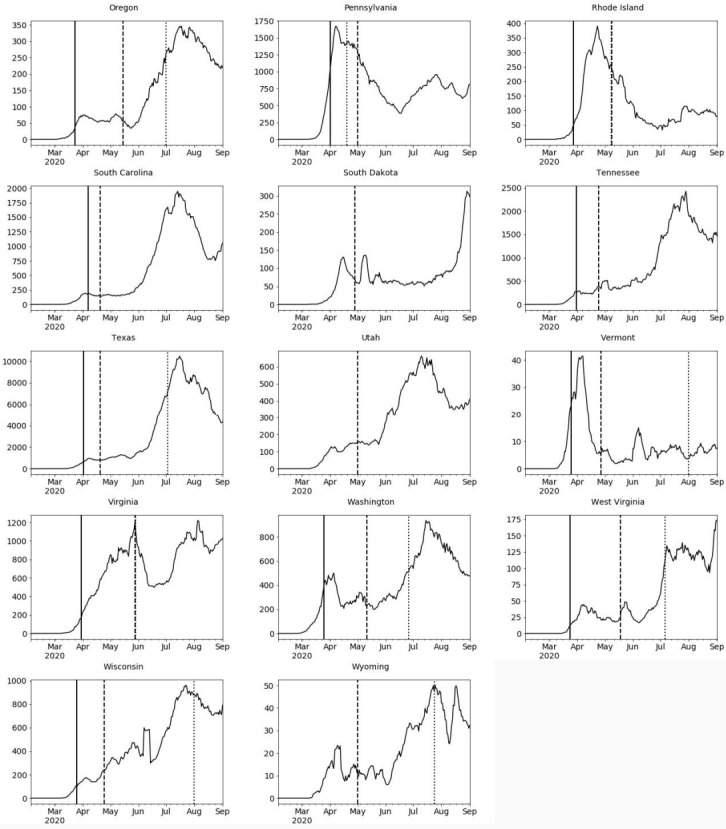
COVID-19 daily confirmed cases (7-day rolling average); US states. Source: author’s analysis of state policy using NY Times [1,26] and CNN data [31].

## Appendix B

**Table A1:** COVID-19 US state policies: reopening and mask mandates; event study model results; March through August 2020 (standardized coefficients).

	Daily Infection Growth Rate ^1^ (%)	
	Std. Coeff.	*p* Value
Daily test growth rate ^1^ (%)	0.07 **	(0.00)
**Reopening implementation (1 = yes) ^2^**		
Pre-policy period		
≥16 days before	0.33 **	(0.00)
11–15 days before	0.00	(0.76)
6–10 days before	−0.00	(0.95)
1–5 days before	−0.00	(0.77)
Reopening date (reference, 0–5 days)		
Post-policy period		
6–10 days after	0.00	(0.88)
11–15 days after	0.01	(0.66)
16–20 days after	0.01	(0.29)
≥21 days after	0.11 **	(0.00)
**Mask mandate implementation (1 = yes) ^3^**		
Pre-policy period		
≥16 days before	0.05 **	(0.00)
11–15 days before	−0.01	(0.59)
6–10 days before	−0.02	(0.08)
1–5 days before	−0.01	(0.21)
Mask mandate date (reference, 0–5 days)		
Post-policy period		
6–10 days after	−0.03 *	(0.01)
11–15 days after	−0.03 *	(0.01)
16–20 days after	−0.02 *	(0.03)
≥21 days after	−0.06 **	(0.00)
Medicaid expansion ^4^ (1 = yes)	−0.01	(0.44)
Percentage essential workers ^5,6^	0.03 *	(0.03)
Percentage minorities ^6^	0.02 *	(0.04)
N	8450	
Log likelihood	−1249.20	
Pseudo R	0.11	

Source: ^1,2^ NY Times [1,26], 2020. ^3^ CNN [31], 2020. ^4^ KFF [14], 2020. ^5^ CBPP [40], 2020. ^6^ ACS [41] (2014–2018). Note: * *p* < 0.05, ** *p* < 0.01.
